# Acute retinal necrosis during the systemic use of Janus kinase inhibitor

**DOI:** 10.1186/s40942-025-00756-4

**Published:** 2025-12-04

**Authors:** Daisuke Watanabe, Kenichi Namba, Keitaro Hase, Kayo Suzuki, Yo Ogino, Michiyuki Saito, Daiju Iwata, Kazuomi Mizuuchi, Miki Hiraoka, Mao Narita, Nobuyoshi Kitaichi, Norihiko Kitaya, Susumu Ishida

**Affiliations:** 1 Department of Ophthalmology, Faculty of Medicine, Graduate School of Medicine, Hokkaido University, N-15, W-7, Kita-ku, Sapporo, 060-8638 Japan; 2 Department of Ophthalmology, Health Sciences University of Hokkaido, Sapporo, Japan; 3 Department of Ophthalmology, Oji General Hospital, Tomakomai, Japan

## To the editor

With the rising use of Janus kinase (JAK) inhibitors worldwide for multiple indications, we wish to alert the readers of the potential of viral retinitis in patients using these agents.

## Case

A 48-year-old man had undergone allogeneic hematopoietic stem cell transplantation one year and five months ago for B-cell lymphoblastic lymphoma and remained in remission. Seven months before presentation, he developed pulmonary graft-versus-host disease (GVHD) and had been treated with oral JAK inhibitor (ruxolitinib 20 mg/day) and prednisolone (11 mg/day). Seven months after initiation of the JAK inhibitor, the patient noted visual field impairment in the left eye. He reported no redness, ocular pain or other ocular symptoms. He was referred to our hospital about one month after the onset of his symptoms.

**Fig. 1:**
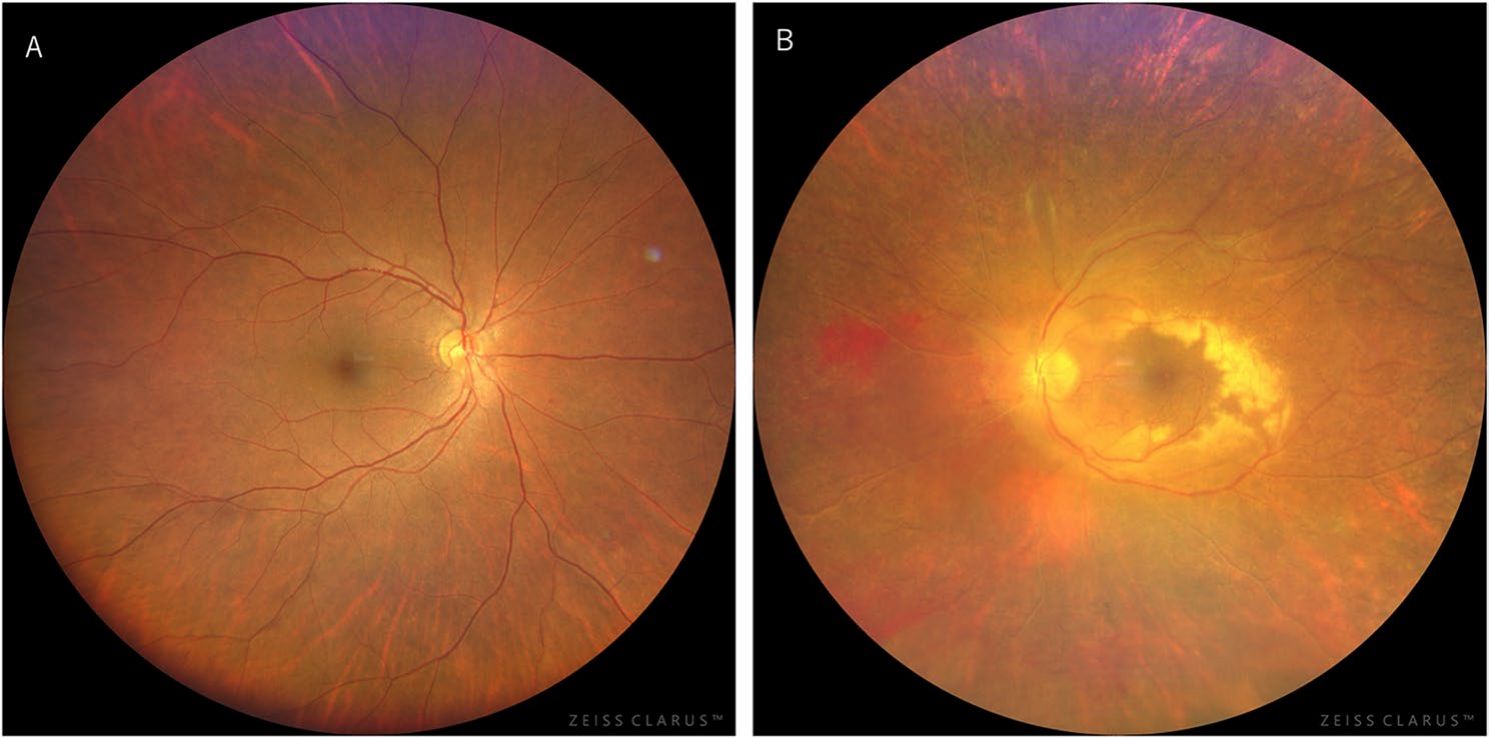
Fundus photographs at initial presentation. The right eye showed no abnormal findings (**A**), whereas the left eye showed retinal hemorrhages, retinal exudates, and widespread white vessels (**B**)

**Fig. 2:**
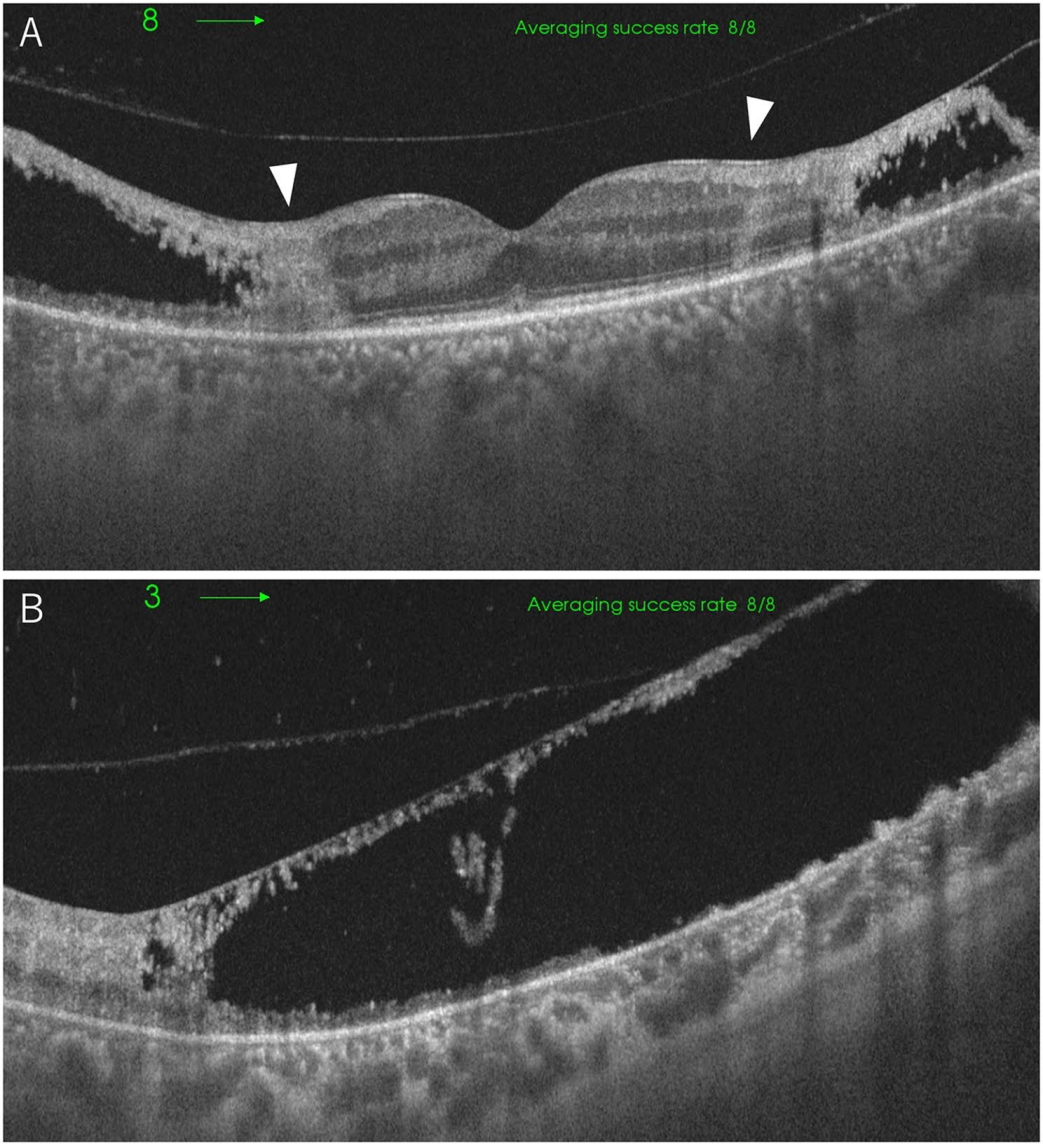
OCT of the left eye at initial presentation. The disruption of the retinal layer and hyperreflective changes (white arrowhead) at the macular area (**A**) and widespread retinal thinning and retinoschisis were observed outside the macula (**B**)

**Fig. 3:**
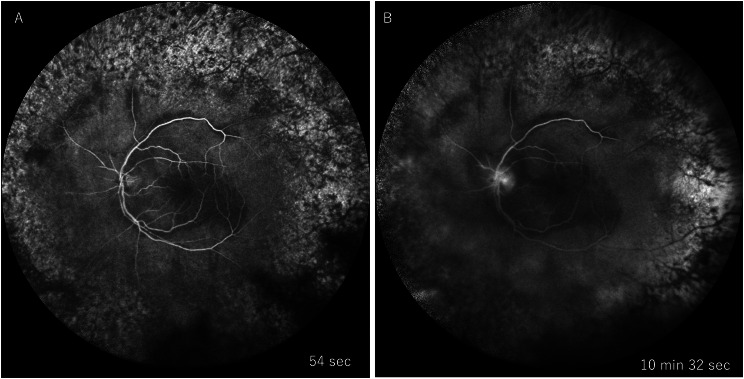
FA findings at initial presentation. In the early phase (54 s) (**A**), whole areas of outside the arcade vessels became non-perfused. There was no vascular leakage during the early to late phases (10 min 32 s) (**B**)

On initial examination, decimal visual acuity was 1.0 (1.5 x S + 0.50 D) in the right eye and 0.8 (1.0 x C -0.50 D Ax 10˚) in the left eye, with intraocular pressure of 18 mmHg OD and 10 mmHg OS. Slit-lamp examination revealed no abnormal findings in the right eye, while the left eye showed a few fine keratic precipitates in the inferior cornea and 2 + flare and 2 + cells in the anterior chamber. Fundus examination was normal in the right eye (Fig. 1-A). In the left eye, there was mild diffuse vitreous opacity, retinal hemorrhages, retinal exudates only in the posterior pole, and widespread white vessels outside the arcade (Fig. 1-B). Optical coherence tomography (OCT) of the left eye showed disruption of the retinal layer and hyperreflective changes with widespread retinal thinning and retinoschisis outside the macula (Fig. 2). Fluorescein angiography revealed that whole areas of outside the arcade vessels became almost non-perfused and showed widespread window defect areas in the periphery, and there was no vascular leakage during the early to late phases (Fig. 3). Due to the following fact that the patient was given immunosuppressants including JAK inhibitor for GVHD, and that anterior chamber and vitreous inflammations were mild, cytomegalovirus (CMV) retinitis was initially suspected. To confirm the diagnosis, blood and aqueous humor tests were performed.

**Fig. 4:**
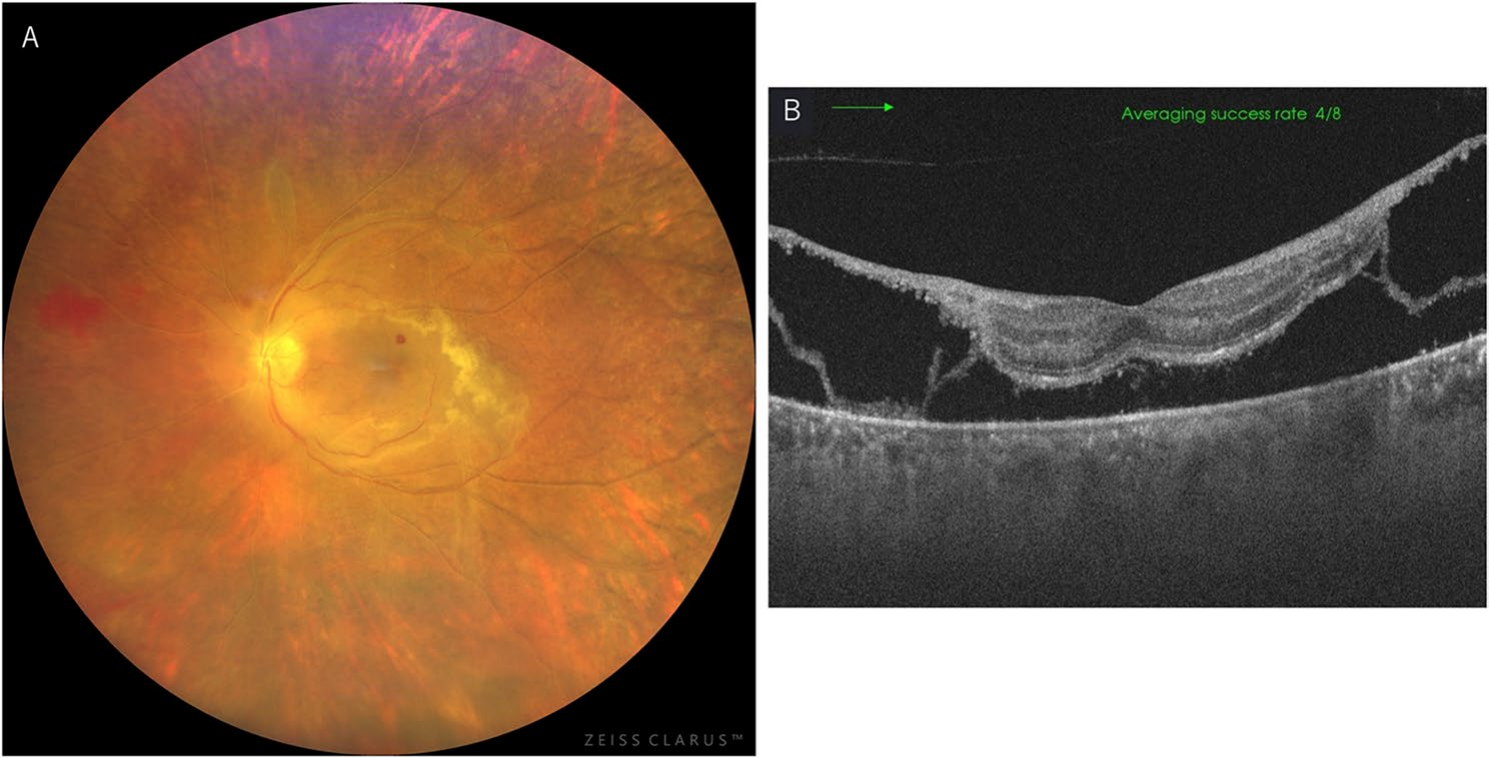
Ocular findings in the left eye four days after the initial presentation. Retinal exudates spreading toward the fovea and further extended white vessels were observed (**A**). On OCT, retinal detachment, including the macula, was seen (**B**)

Four days later, visual acuity in the left eye had decreased to 0.5 and the retinal exudates spread toward the fovea and white vessels also extended (Fig. 4-A), while OCT revealed retinal detachment involving the fovea (Fig. 4-B). Blood tests showed negative C7HRP (an indicator of CMV antigenemia) but high VZV-IgM and VZV-IgG. PCR examination with aqueous humor was positive for VZV-DNA and negative for CMV and HSV, leading to a diagnosis of ARN; however, given its rapid progression and the underlying immunosuppression, it was considered to be progressive outer retinal necrosis (PORN). The patient was admitted and treated with intravitreal ganciclovir injection (2.0 mg) and intravenous acyclovir (30 mg/kg/day). Subsequently, pars plana vitrectomy (PEA + PPV + endolaser photocoagulation + silicone oil tamponade + sub-Tenon triamcinolone acetonide + encircling) was performed.

**Fig. 5:**
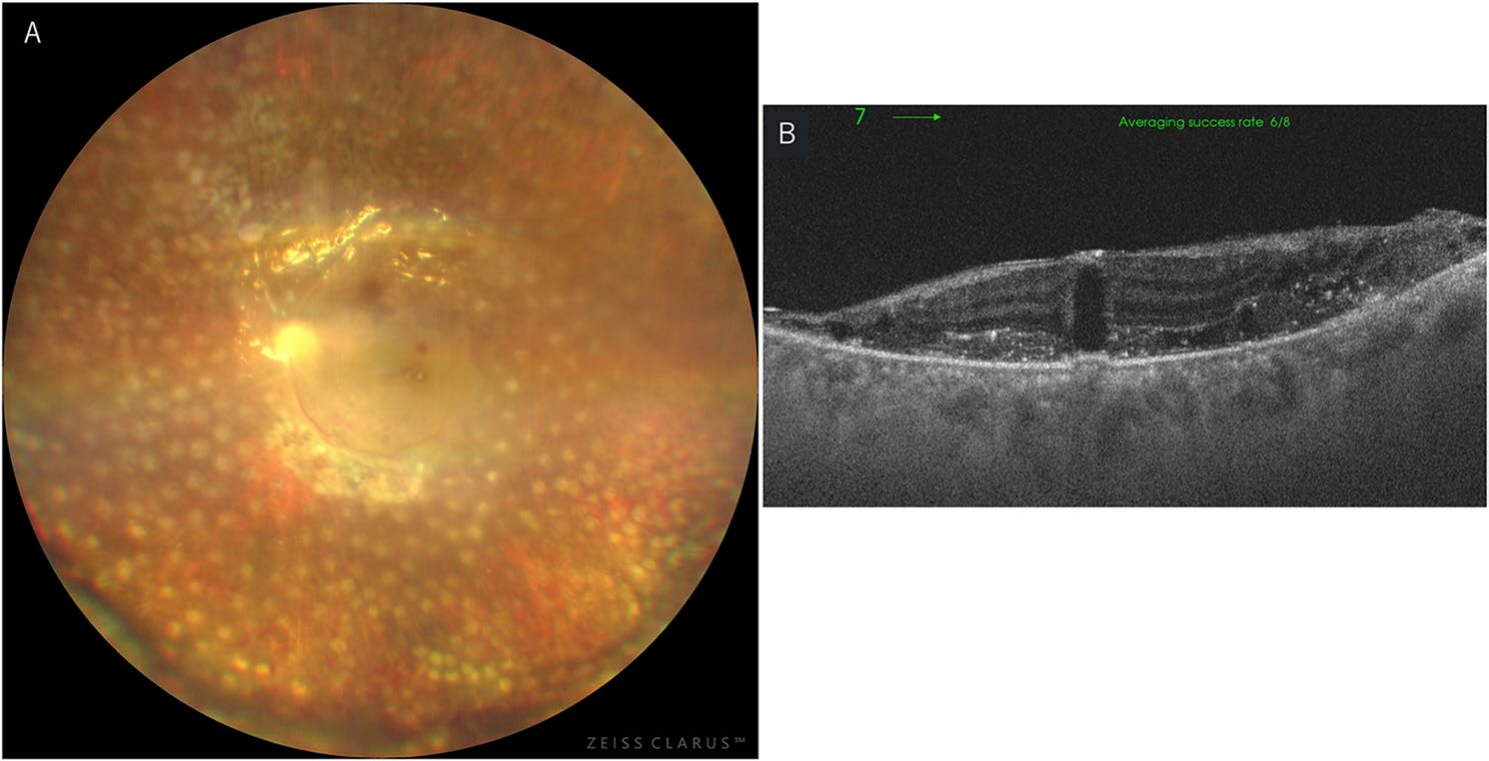
Postoperative ocular findings in the left eye. The white dots are laser photocoagulation scars. Although the retinal exudates have resolved, the macula has become slightly opaque (**A**). On OCT, although the retina was successfully reattached, the outer layer of retina was disrupted (**B**)

Postoperative findings are shown in Fig. 5. Although the retina was successfully reattached, final visual acuity in the left eye was limited to light perception. The patient has since maintained light perception without progression to phthisis bulbi.

## Discussion

Since the approval of tofacitinib in the United States in 2012 for the treatment of rheumatoid arthritis, various JAK inhibitors have been developed and are now widely used in the world. The JAK-signal transducer and activator of transcription (STAT) pathway is a key intracellular signaling cascade involved in immune regulation and hematopoiesis [1]. The JAK family consists of four members – JAK1, JAK2, JAK3, and TYK2 – that transmit signals from various cytokines through distinct combinations. By blocking these kinases, JAK inhibitors suppress proinflammatory cytokines and exert potent immunosuppressive effects.

Currently, more than ten types oral JAK inhibitors are used worldwide for the treatment of a wide range of conditions, including rheumatoid arthritis, inflammatory bowel disease, atopic dermatitis, ankylosing spondylitis, psoriasis, alopecia areata, myelofibrosis, polycythemia vera, giant cell arteritis [2] and so on. Notably, JAK inhibitors have also been investigated for the treatment of ocular noninfectious uveitis [5]. Their adoption has been rapid, with the global market estimated at approximately 25 billion USD in 2025 and expected to grow to 47 billion USD by 2029 [6].

However, JAK inhibitors are associated with an increased risk of opportunistic infections [1–4]. Approximately 5% of patients develop reactivation of VZV, most commonly presenting as herpes zoster, but occasionally leading to severe complications such as VZV meningoencephalitis [4]. Other adverse events have been reported include cardiovascular, renal, and hepatic dysfunction. These reports highlight the need for careful monitoring during treatment with JAK inhibitors.

In the present case, the patient had been treated with ruxolitinib (Jakavi®), a JAK1/JAK2 inhibitor, which had recently been approved in Japan for the treatment of GVHD following hematopoietic stem cell transplantation. Considering the known risk of VZV reactivation, ruxolitinib may have contributed to the development of VZV-associated PORN in this patient. Furthermore, the profound anti-inflammatory effect of JAK inhibition may have suppressed intraocular inflammation, thereby allowing retinal necrosis to progress rapidly with minimal inflammatory signs, indicative of the clinical course of PORN.

To the best of our knowledge, ARN or PORN has not previously been reported in association with JAK inhibitor therapy. Given the potential for these devastating, vision-threatening complications, patients receiving JAK inhibitors should be counseled regarding the risk of ocular symptoms. Prompt ophthalmic evaluation is warranted if sudden visual decline, visual field defects, blurred vision, and/or ocular redness occur. Continued vigilance is needed to determine whether similar cases emerge in the future.

## Data Availability

Data is available from the corresponding author on reasonable request.

## Abbreviations

ARN: Acute retinal necrosis
PORN: Progressive outer retinal necrosis
JAK: Janus kinase
STAT: Signal transducer and activator of transcription
VZV: Varicella zoster virus
CMV: Cytomegalovirus
HSV: Herpes simplex virus
GVHD: Graft versus host disease
OCT: Optical coherence tomography
